# Ibrutinib versus allogeneic haematopoietic stem cell transplant in relapsed/refractory del(17p) chronic lymphocytic leukaemia/small lymphocytic lymphoma

**DOI:** 10.1111/bjh.70803

**Published:** 2026-08-31

**Authors:** Farrukh T. Awan, Ran Liu, Hsin‐Hui Huang, Matthew Bye, Kwang Woo Ahn, Gabriel Krigsfeld, Anat Raz, Heather Wolfe, Mehdi Hamadani

**Affiliations:** ^1^ University of Texas Southwestern Medical Center Dallas Texas USA; ^2^ AbbVie North Chicago Illinois USA; ^3^ Center for International Blood and Marrow Transplant Research (CIBMTR) Medical College of Wisconsin Milwaukee Wisconsin USA; ^4^ Division of Biostatistics Medical College of Wisconsin Milwaukee Wisconsin USA; ^5^ Division of Hematology & Oncology, Department of Medicine Medical College of Wisconsin Milwaukee Wisconsin USA

**Keywords:** allogeneic haematopoietic stem cell transplantation, chronic lymphocytic leukaemia, del(17p)

## Abstract

After adjusting for confounders, the ibrutinib cohort had significantly better overall survival (adjusted hazard ratio, 0.39; 95% confidence interval, 0.23–0.68; *p* = 0.0008) than the allogeneic haematopoietic stem cell transplantation (alloHSCT) cohort. Our large, retrospective analysis suggests that ibrutinib treatment may offer improved overall survival and progression‐free survival compared to alloHSCT in patients with relapsed/refractory del(17p) chronic lymphocytic leukaemia/small lymphocytic lymphoma.

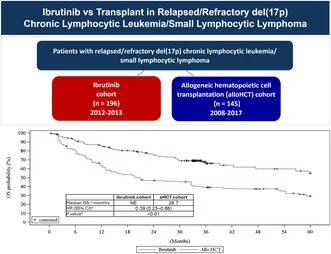

To the Editor,

Prognosis and treatment outcomes are inferior for patients with chronic lymphocytic leukaemia (CLL)/small lymphocytic lymphoma (SLL) with del(17p) and *Tp53* mutations compared with patients without these abnormalities.^1^ Historically, these patients, if fit and without advanced comorbid conditions, would undergo allogeneic haematopoietic stem cell transplantation (alloHSCT) early in the disease course,^2^ given the limited activity and duration of response with historic chemoimmunotherapy or antibody‐based regimens like fludarabine, cyclophosphamide and rituximab^3^ or alemtuzumab.^4^ These recommendations were primarily derived from small studies of relatively young patients (median age in the mid‐50s) and included only a limited number of patients with del(17p). These studies demonstrated a 5‐year progression‐free survival (PFS) of approximately 40% with alloHSCT, with reduced‐intensity conditioning (RIC) established as the preferred conditioning approach.^5^, ^6^ However, alloHSCT was limited to a small number of patients with CLL, given the median age of diagnosis of 72 years, lack of suitable donors (era predating routine use of alternative donors), and difficulty in controlling chemorefractory disease.^5^, ^6^

The introduction of the first‐in‐class Bruton tyrosine kinase (BTK) inhibitor, ibrutinib, significantly improved outcomes for patients with relapsed/refractory (R/R) CLL/SLL, including those with high‐risk genetic features such as del(17p).^7^, ^8^ The RESONATE‐17 study, the largest prospective clinical trial of 144 patients with relapsed CLL and del(17p), with a median of two prior lines of therapy, demonstrated a 2‐year PFS of 63% and overall survival (OS) of 75%.^9^ With tolerable adverse events and the option to treat older patients with significant comorbidities, this was a major advance for patients with high‐risk disease who previously had limited options. As a result of the efficacy of ibrutinib, alloHSCT was only considered suitable for patients who had progressed after targeted therapies, including BTK inhibitors.^2^ While this was the gradual, natural progression of treatment advances and patient dynamics, it was not supported by clear data, especially for patients who would ordinarily be considered candidates for alloHSCT.

Herein, we report the results of the most extensive comparative analysis of patients with R/R CLL/SLL with concurrent del(17p), drawing from the retrospective alloHSCT database of the Centre for International Blood and Marrow Transplant Research (CIBMTR) and the large prospective clinical trials of ibrutinib.

We compared alloHSCT with ibrutinib therapy in patients with R/R del(17p) CLL/SLL. This retrospective analysis used data from the CIBMTR registry over a 10‐year period and compared them with data from the randomized Phase 3 RESONATE clinical trial (NCT01578707) and the open‐label Phase 2 RESONATE‐17 study (NCT01744691).^9^ Adults with R/R CLL/SLL with del(17p) received either alloHSCT (reported to CIBMTR during 2008–2017) or single‐agent ibrutinib (420 mg/day; enrolment dates: RESONATE, June 2012–April 2013; RESONATE‐January 17 2013–June 2013). Additional inclusion criteria for patients were Eastern Cooperative Oncology Group (ECOG) Performance Status score of 0 or 1, adequate organ function, and ≤5 prior lines of therapy, as previously published.^8^, ^9^, ^10^ Patients in the alloHSCT cohort were included in the analysis if they received alloHSCT from a human leucocyte antigen‐identical related donor, a mismatched related donor or a matched or mismatched unrelated donor. All sources of grafts, all conditioning regimens, and all graft‐versus‐host disease (GVHD) prophylactic approaches were permitted. Patients were excluded if they received syngeneic donor transplantation or prior ibrutinib therapy.

The baseline characteristics and disease features were compared between the two cohorts. The primary outcomes measured for patients who received alloHSCT or ibrutinib were OS and PFS. Additionally, for patients undergoing alloHSCT, the cumulative incidence of relapse/progression, non‐relapse mortality (NRM) and GVHD were reported. Survival outcomes were analysed using the Kaplan–Meier method and the Cox proportional hazards model. A detailed description of the statistical methods is provided in the Supporting Information S1.

The median follow‐up was capped at 60 months for the alloHSCT cohort (*n* = 145). The median follow‐up for ibrutinib‐treated patients (*n* = 196) was 64 months on the RESONATE (*n* = 53) and RESONATE‐17 (*n* = 143) clinical trials respectively. The median age among patients in the alloHSCT versus ibrutinib cohort was 58.9 vs. 64.5 years (*p* < 0.001), and 70% vs. 34% (*p* < 0.001) had an ECOG performance status score of 0 respectively. The median time from diagnosis to treatment was 42 vs. 70 months (*p* < 0.001), and the proportion of patients with bulky disease was 21% vs. 53% (*p* < 0.001) in the alloHSCT and ibrutinib cohorts respectively. In patients who underwent alloHSCT, 77% received reduced‐intensity (RIC) or non‐myeloablative (NMA) conditioning, 83% received calcineurin inhibitors with mycophenolate mofetil or methotrexate as GVHD prophylaxis, and 29% received anti‐thymocyte globulin (Table 1).

**TABLE 1:** Patient demographics.

Characteristic	Ibrutinib cohort, *n* = 196	alloHSCT cohort, *n* = 145	*p*‐value
Age, median (range), years	65 (30–89)	59 (32–73)	<0.01
Women, *n* (%)	67 (34)	47 (32)	0.73
ECOG 0–1, *n*/*N* (%)	195/196 (99)	134/137 (98)	<0.01
Race, *n* (%)			0.06
White	177 (90)	124 (86)	
Black or African American	10 (5)	17 (12)	
Rai stage 3–4, *n* (%)	123/196 (63)	31/106 (29)	<0.01
Additional cytogenetic abnormalities, *n*/*N* (%)			<0.01
del(13q)	125/188 (66)	75/123 (61)	0.32
del(11q)	34/194 (18)	30/145 (21)	0.46
Trisomy 12	36/182 (20)	25/145 (17)	0.56
Time from diagnosis to treatment <1 year, *n* (%)	182 (93)	123 (85)	0.02
Number of prior treatments ≥2, *n* (%)	136 (69)	110 (76)	<0.01
Bulky disease ≥5 cm, *n*/*N* (%)	102/194 (53)	26/124 (21)	<0.01
Conditioning regimen, *n*/*N* (%)			
Myeloablative	NA	21/145 (14)	NA
RIC/NMA	NA	111/145 (77)	
Not reported	NA	13/145 (9)	
GVHD prophylaxis, *n*/*N* (%)			NA
None	NA	1/144 (1)	
CNI + MMF^b^	NA	59/144 (41)	
CNI + MTX^b^	NA	62/144 (43)	
Others	NA	22/144 (15)	

Abbreviations: alloHSCT, allogeneic haematopoietic stem cell transplantation; CNI, calcineurin inhibitor; ECOG, Eastern Cooperative Oncology Group Performance Status; GVHD, graft‐versus‐host disease; MMF, mycophenolate mofetil; MTX, methotrexate; NA, not applicable; NMA, non‐myeloablative; RIC, reduced‐intensity conditioning.

At 36 months, in the alloHSCT and ibrutinib cohorts, respectively, the PFS were 33% (95% confidence interval [CI], 19–47) and 55% (95% CI, 47–62); and OS rates were 39% (95% CI, 23–55) and 66% (95% CI, 58–73) (Figure 1A,B). After adjusting for confounders, the ibrutinib group had significantly better PFS (adjusted hazard ratio [HR], 0.43; 95% CI, 0.27–0.69; *p* = 0.0005) and OS (adjusted HR, 0.39; 95% CI, 0.23–0.68; *p* = 0.0008) versus the alloHSCT group.

**FIGURE 1:**
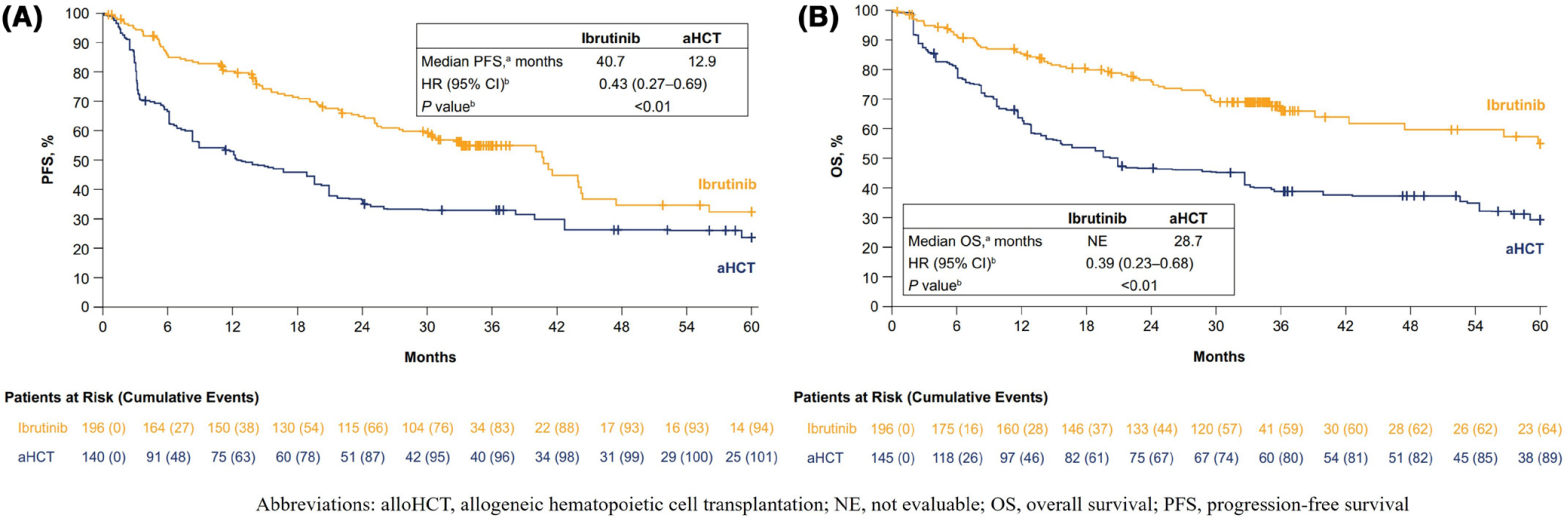
(A) Progression‐free survival outcomes. (B) Overall survival outcomes. alloHSCT, haematopoietic stem cell transplantation; NE, not evaluable; OS, overall survival; PFS, progression‐free survival.

Causes of death varied; 69 patients (47.1%) in the alloHSCT cohort and 37 patients (32.9%) in the ibrutinib cohort died. The most common cause of death was disease‐related in both cohorts (30% vs. 19% in the alloHSCT and ibrutinib cohorts, respectively) (Table S1). In the alloHSCT cohort, the 5‐year cumulative incidence of relapse was 36.4% (95% CI, 0.29–0.45), NRM was 35.7% (95% CI, 0.29–0.45), and chronic GVHD was 66.2% (95% CI, 0.59–0.74) (Figures S1–S3).

The advancement of therapeutic options for patients with CLL over the last decade has led to a gradual decline of alloHSCT even in patients with high‐risk del(17p) disease. Our large retrospective analysis is the first study to provide data supporting the gradual, organically occurring change in practice and to demonstrate that ibrutinib treatment improves PFS and OS outcomes compared with alloHSCT in patients with relapsed/refractory (R/R) del(17p) CLL/SLL. These data will provide the basis for definitive, evidence‐based recommendations to use alloHSCT only after BTK failure.

The alloHSCT cohort was limited to 2008–2017 to minimize the cohort effect, ensure patients had access to consistent supportive care across both cohorts, and allow for adequate follow‐up of the latest patient analysed; however, an argument can be made that recent advances in conditioning therapy and the use of post‐transplant cyclophosphamide may result in improved outcomes for the alloHSCT cohort. Moreover, curtailing follow‐up to 60 months may miss a subset of patients who experienced sustained remission following alloHSCT. Additionally, data on subsequent therapies or depth of remission at the time of alloHSCT are not routinely available in the CIBMTR cohort, hence limiting those analyses.

Our results compare favourably to those reported in the CLL3X study of 90 patients with R/R CLL who underwent alloHSCT.^6^, ^11^ Of the 78 patients with genetic data, only 13 (17%) had del(17p). Seven of these patients were alive after a median follow‐up of 43 months, corresponding to 4‐year event‐free survival and OS of 45% and 59% respectively. Another study of 108 patients who underwent alloHSCT consecutively at a single centre included only 13 patients with del(17p) and reported 5‐year PFS and OS of 46% and 67% respectively.^5^

Other large retrospective studies have established RIC as the preferred regimen, given its improved PFS and OS and lower transplant‐related mortality. However, their reporting of patient outcomes with del(17p) was limited. Additionally, all these studies had a median patient age in the mid‐50s, with a younger median among patients who underwent myeloablative conditioning, limiting their applicability to most patients with CLL.

In early studies of ibrutinib, the median PFS in patients with R/R del(17p) CLL was 28 months, representing a significant improvement over previous therapeutic options, including chemoimmunotherapy, which had a median PFS of 11 months. An integrated analysis of 230 R/R CLL/SLL patients with del(17p) was also performed and demonstrated a 13‐month PFS of 57% and an OS of 69%. These patients received a median of two prior lines of therapy and had a median age of 65, with 37% aged 70 or older.^12^

Due to clinical trial enrolment criteria, patients in the ibrutinib cohort were older, had more comorbidities, and had a longer time from diagnosis than those in the alloHSCT cohort. This was rigorously controlled using average treatment effect on the treated, inverse propensity score weighting (ATT‐IPSW), a propensity score model‐based analysis described in detail in the supplement.

In the current era, patients are considered for alloHSCT only after failing conventional treatment for CLL, including BTK inhibitors. Our study now provides the most extensive comparative evidence of ibrutinib's superiority over alloHSCT for PFS and OS in patients with R/R CLL and del(17p). This further justifies using BTK inhibitors for patients with high‐risk disease before considering alloHSCT, mainly because data support alloHSCT in patients with well‐controlled disease that relapses after BTK inhibitor therapy, especially given the excellent outcomes observed with these agents.^13^, ^14^, ^15^

## AUTHOR CONTRIBUTIONS

**Ran Liu:** Investigation; writing – original draft; supervision; data curation; software; formal analysis; project administration; visualization; writing – review and editing; validation. **Matthew Bye:** Investigation; writing – original draft; writing – review and editing; formal analysis; project administration; resources; methodology; software. **Hsin‐Hui Huang:** Investigation; writing – original draft; writing – review and editing; validation; visualization; formal analysis; software; project administration; supervision; data curation; methodology. **Mehdi Hamadani:** Conceptualization; investigation; writing – original draft; writing – review and editing; visualization; validation; methodology; funding acquisition; formal analysis; project administration; resources; supervision; data curation. **Kwang Woo Ahn:** Investigation; writing – original draft; writing – review and editing; validation; visualization; methodology; formal analysis; project administration; software; resources. **Gabriel Krigsfeld:** Investigation; writing – original draft; writing – review and editing; project administration; resources; formal analysis. **Anat Raz:** Writing – original draft; investigation; writing – review and editing; project administration; formal analysis; supervision; resources. **Heather Wolfe:** Investigation; writing – original draft; writing – review and editing; supervision. **Farrukh T. Awan:** Conceptualization; investigation; funding acquisition; writing – original draft; methodology; validation; visualization; writing – review and editing; project administration; formal analysis; data curation; supervision; resources; software.

## FUNDING INFORMATION

AbbVie funded this study and participated in its design, research, analysis, data collection, interpretation and review and approval of the publication. No honoraria or payments were made for authorship.

## CONFLICT OF INTEREST STATEMENT

FTA has provided consultancy to Loxo Oncology, BeiGene, Dava Oncology, AstraZeneca, Genmab, Adaptive Biotechnologies, Bristol Myers Squibb, AbbVie, Incyte, Miltenyi, Invivyd and Pierre Fabre, and has received research funding from AbbVie/Pharmacyclics and serves on the DSMC for Ascentage, Caribou Biosciences and AstraZeneca. MH has provided consultancy to Genmab, CRISPR, Allovir, Caribou Biosciences, Autolus, Forte Biosciences, Byondis, Kite, Daiichi Sankyo and Myeloid Therapeutics. Research Support/Funding: ADC Therapeutics; Spectrum Pharmaceuticals; Astellas Pharma. Speaking: AstraZeneca, ADC Therapeutics, BeiGene, Kite, Sobi. RL, HHH, GK, AR: are full‐time employees of AbbVie Inc. and hold stock or stock options.

## ETHICS STATEMENT

Informed consent was obtained from patients enrolled to all the ibrutinib trials and CIBMTR database after specific institutional and central IRB approvals.

## PATIENT CONSENT STATEMENT

Specific patient consent was not required for a retrospective, blinded analysis of existing datasets.

## Supporting information


**Table S1.** Causes of death by cohort.
**Figure S1**. Cumulative incidence of relapse in patients undergoing allogeneic haematopoietic stem cell transplantation.
**Figure S2**. Cumulative incidence of non‐relapse mortality in patients undergoing allogeneic haematopoietic stem cell transplantation.
**Figure S3**. Cumulative incidence of chronic graft‐versus‐host disease in patients undergoing allogeneic haematopoietic stem cell transplantation.

## Data Availability

CIBMTR makes its publication analysis datasets freely available to the public for secondary analysis while safeguarding the privacy of participants and protecting confidential and proprietary data: https://cibmtr.org/CIBMTR/Resources/Publicly‐Available‐Datasets.
