# Supplementary material for: Ibrutinib versus allogeneic haematopoietic stem cell transplant in relapsed/refractory del(17p) chronic lymphocytic leukaemia/small lymphocytic lymphoma
Source: Br J Haematol. 2026 Aug 31;209(5):2284–8. doi: 10.1111/bjh.70803 (PMC13685468; doi:10.1111/bjh.70803)
Supplement: Supplementary file 1 [file BJH-209-2284-s001.docx]

# **Supplementary Materials**

This material accompanies the paper “Ibrutinib vs Transplant in Relapsed/Refractory del(17p) Chronic Lymphocytic Leukemia/Small Lymphocytic Lymphoma.”

**Additional Statistical Methods:**

CIBMTR is a research collaboration between the Medical College of Wisconsin and the National Marrow Donor Program (NMDP). More than 350 medical centers worldwide submit clinical data about HCT and cellular therapies to CIBMTR. Data are submitted before infusion and at 3 months, 6 months, and yearly thereafter. CIBMTR ensures data quality and safeguards participant confidentiality. The NMDP Institutional Review Board reviews CIBMTR’s research. Patients or guardians give informed consent for research.

For continuous variables with a normal distribution, p-values were calculated using a two-sample t-test. A Mann-Whitney U test was employed to assess non-normally distributed variables. For categorical variables with expected cell count(s) n <5 and n ≥5, p-values were calculated using Fisher's Exact and Chi-squared tests, respectively. Outcome comparisons were adjusted by the average treatment effect on treated with inverse propensity score probability weighting (ATT-IPSW) to balance key confounders: age, sex, race, bulky disease, Rai stage, prior treatment, ECOG performance status, time from diagnosis to treatment, del(11q), del(13q), and trisomy 12. A sensitivity analysis was conducted using an E-value to assess the minimum strength of association that an unmeasured cofounder would need to impact the treatment and outcome to explain a treatment-outcome association. The assumption of IPSW, “no unmeasured cofounder would need to impact the treatment and outcome to explain treatment-outcome association confounders,” was assessed using E-value as the sensitivity analysis. Sensitivity analysis for unmeasured confounding factors performed on the full analysis set yielded an E-value of 2.95 (95% CI, 1.86–4.35), indicating that considerable unmeasured confounding would be required to negate the effect estimate.

The average treatment effect on the treated inverse propensity score weighting (ATT-IPSW) method, a propensity score model-based analysis, was applied to mitigate this imbalance, and a multiple imputation method was used to account for missing baseline covariate data in both cohorts. While unmeasured confounders at baseline for the ATT-IPSW method may exist, sensitivity analyses indicated that only relatively strong unmeasured confounders would nullify the observed treatment effects. This was confirmed by calculating an E-value to assess the minimum strength of association that an unmeasured confounder would need to have to explain a treatment-outcome association.(1) One limitation of the analysis was that propensity scores with extreme weights were truncated at 10 (the 99th percentile), which can reduce covariate balance and lead to biased estimates of treatment effects.

## **Table S1:** Causes of Death by Cohort

|  | **Ibrutinib cohort**  **No. (%)** | **alloHCT cohort**  **No. (%)** |
| --- | --- | --- |
| No. patients | 196 | 145 |
| No. deaths | 64 (33) | 69 (47) |
| ***Causes of death*** | | |
| Primary disease | 37 (19) | 43 (30) |
| Infection | 15 (8) | 16 (11) |
| GVHD | 1 (1) | 18 (12) |
| Organ failure | 4 (2) | 6 (4) |
| Other | 1 (1) | 8 (6) |
| Hemorrhage | 1 (1) | 3 (2) |
| Not reported | 2 (1) | 2 (1) |
| Cardiovascular | 3 (2) | 0 (0) |
| Interstitial pneumonitis | 0 (0) | 1 (1) |
| Secondary malignancy | 0 (0) | 1 (1) |
| Vascular | 0 (0) | 1 (1) |

Abbreviations: alloHCT, allogeneic hematopoietic cell transplantation; GVHD, graft-vs-host disease

## **Figure S1:** Cumulative Incidence of Relapse in Patients Undergoing Allogeneic Hematopoietic Cell Transplantation

## **Figure S2:** Cumulative Incidence of Non-Relapse Mortality in Patients Undergoing Allogeneic Hematopoietic Cell Transplantation

## **Figure S3:** Cumulative Incidence of Chronic Graft-vs-Host Disease in Patients Undergoing Allogeneic Hematopoietic Cell Transplantation

Abbreviations: alloHCT, allogeneic hematopoietic cell transplantation. NRM, Non-Relapse Mortality

**Supplemental References:**

1.	VanderWeele TJ, Ding P. Sensitivity Analysis in Observational Research: Introducing the E-Value. Ann Intern Med. 2017;167(4):268-74.

2.	Sobecks RM, Leis JF, Gale RP, et al: Outcomes of human leukocyte antigen-matched sibling donor hematopoietic cell transplantation in chronic lymphocytic leukemia: myeloablative versus reduced-intensity conditioning regimens. Biol Blood Marrow Transplant 20:1390-8, 2014

3.	CIBMTR: 2023 Annual Report, 2024

4.	CIBMTR: Data Collection Forms, CIBMTR, 2024

5.	CIBMTR: Protocols and Consent Forms, CIBMTR, 2024

6.	CIBMTR: Protocol For a Research Database for Hematopoietic Cell Transplantation, Other Cellular Therapies and Marrow Toxic Injuries, version 9.1, 2022

7.	CIBMTR: Protocol for a research sample repository for hematopoietic cell transplantation, other cellular therapies and marrow toxic injuries, (ed Version 13.0), CIBMTR, 2021

8.	CIBMTR: Data Protection and Privacy, CIBMTR, 2023

9.	Li F, Morgan KL, Zaslavsky AM: Balancing Covariates via Propensity Score Weighting. Journal of the American Statistical Association 113:390-400, 2018
